# Target Size Manipulations Affect Error-Processing Duration and Success Perceptions but not Behavioural Indices of Learning

**DOI:** 10.3390/brainsci9050119

**Published:** 2019-05-23

**Authors:** Nicole T. Ong, Jamie Hawke, Nicola J. Hodges

**Affiliations:** School of Kinesiology, University of British Columbia, BC V6T 1Z1, Canada; nicole.ong@alumni.ubc.ca (N.T.O.); hawkej@mail.ubc.ca (J.H.)

**Keywords:** motor learning, motor control, throwing, motivation, EMG

## Abstract

We evaluated if and how success perceptions, through target size manipulations, impact processes related to motor learning. This work was based on recent literature suggesting that expectations and self-efficacy exert a direct impact on learning. We measured arousal, kinematics, learner expectancies, motivation, and outcomes in a dart-throwing task. Novices (*n* = 29) were assigned to either a “Large-target” (horizontal target, 10-cm high) or “Small-target” (2-cm high) group for practice (*t* = 90), and both groups completed 24-h retention tests. The Small-target group took longer to plan and process feedback in the pre-throw and post-throw periods, respectively, and showed larger joint amplitudes early in practice compared to the Large-target group. As predicted, the Large-target group made more hits and had heightened outcome expectancies compared to the Small-target group. Surprisingly, only the Large-target group performed better than they expected. Despite the Large-target group having more target hits, enhanced expectancies, and more unexpected success, this group did not outperform the Small-target group on behavioural indices of performance and learning. This research questions assumptions and results related to success-related manipulations for task performance and mechanisms related to target size manipulations.

## 1. Introduction

Enhanced expectancies of success have been shown to positively impact motor learning, which has led researchers to propose a significant role for affective and/or socio-cognitive processes in theories of motor learning [1]. Until recently, the prevalent view on affective and socio-cognitive processes was that they influenced motor learning through indirect means. It was thought that practice manipulations promoting positive affect or heightened expectancies resulted in greater motivation, so that learners exerted more time and effort in further practice. This view has been challenged in studies where enhanced expectancies or success perceptions were shown to have a direct and immediate positive impact on motor learning, whilst controlling for the amount of practice received by learners. 

### 1.1. Evidence that Success-Enhancing Manipulations Benefit Learning

Enhanced success perceptions have benefited motor learning in various scenarios. False, yet positive social comparative feedback, in comparison to negative feedback [2,3,4,5,6], or selective feedback on practice trials with better versus worse outcomes [7,8,9] have yielded motor learning benefits. In learning to maintain balance on a stabilometer (a raised, unstable platform), participants in a positive social comparative feedback group received—in addition to their true error scores—false feedback that group averages were 20% worse than their true score [4]. A control group received only true error scores, and a negative feedback group received true error scores and false feedback that group averages were 20% better than their scores. Across practice and retention phases, the positive feedback group outperformed the control and negative groups, although all groups received the same amount of practice and veridical feedback about actual error [4].

The typical protocol for selectively providing feedback in motor learning studies involves withholding outcome feedback by occluding vision. To enhance success perceptions, outcome feedback on the better half of the trials performed in a practice block, compared to the worse half of the trials, is provided [8,9,10]. Similar to effects associated with social comparative feedback, superior performance has been reported for the positive (best trials) feedback groups versus the negative (worst trials) feedback groups in practice [9,10] and/or retention [8]. Researchers have suggested that these advantages are linked to the greater self-efficacy and confidence associated with more positive performance feedback.

Another success manipulation that researchers have employed in an attempt to enhance learning is through targets that are easier to hit [11] or criteria for success that is easier to achieve [12,13]. In a golf-putting study, participants who aimed at a large (easier) target compared to a small (more difficult) target showed enhanced accuracy in relation to the target centre, in both practice and delayed retention [11].

### 1.2. Potential Mechanisms for Success Perception Effects on Motor Learning

The learning advantages associated with success manipulations have been attributed to various factors. The first is an increase in motivation due to heightened expectancies, which potentially promotes effort and concentration within the practice session [1]. Increased effort has been indexed by performers’ ratings of perceived effort, or through physiological measures of arousal, such as electrodermal activity (EDA) and heart rate. Improved concentration on the task not only prevents distractions, but likely encourages a more optimal attentional focus [1]. Related to attentional focus, a second factor thought to contribute to success manipulations effects is cognitive processing. When errors are minimised in practice, learners may be less inclined to engage in hypotheses testing and therefore accrue less explicit knowledge about how to perform [14]. Enhanced expectancies are likely to bring about a similar reduction in cognitive processing. Having less explicit task-relevant knowledge has been linked with more robust motor learning, promoting greater automaticity [15]. This more implicit mode of learning has been shown to prevent performance decrements in retention and under conditions that demand cognitive resources, including dual tasks and competitive stress [14,16]. A third process proposed to explain motor learning benefits associated with success manipulations is memory consolidation. Consolidation is modulated by dopamine, which facilitates stable memory formation during the retention period when practice has ceased; this is also referred to as offline learning [17,18]. Consolidation is enhanced when individuals experience rewards in practice, and this is thought to be due to reward-associated biochemical processes occurring during sleep or rest [17]. When the effects of success manipulations are delayed (i.e., only seen in retention but not practice), it is likely that processes associated with consolidation are responsible [12,13]. 

### 1.3. Evidence that Success-Enhancing Manipulations Do Not Benefit Motor Learning

Despite empirical evidence and hypothesised mechanisms to explain how success-enhancing manipulations benefit motor learning, there have been a number of studies failing to show group differences associated with manipulations to success. Importantly, these ‘failure to replicate’ studies have shown increases in confidence or perceptions of success, in the absence of any behavioural impacts on performance or learning. For example, in two studies where selective feedback was provided for trials with better versus worse outcomes, no group differences were shown in either practice or retention [19,20]. This was despite more positive judgments of learning reported by the groups that received better-outcomes feedback [19].

In a dart-throwing study where target size was manipulated, no practice or retention differences were shown between a large and small target group, despite elevated success rates and self-efficacy in the large versus small target group [21]. In the earlier described golf-putting study [11], group differences were apparent from the very first practice block (in addition to a trend in the pretest), raising concerns about individual differences explaining later effects, rather than success perceptions. There are also motor control-related reasons that would lead to questions concerning whether target size differences are related to strategies in control. Movements to small rather than large targets differ in their control due to established relations between variability of movement, forces, and movement duration [22]. With a smaller target, greater precision demands would likely lead to more time in movement planning (preparation time) as well as longer duration movements than practice with larger targets. Thus, movement kinematics would look different and be potentially more variable for targets that require more precision. To date, movement process measures have generally been missing from studies of success manipulations, even though strategy differences in execution and planning might explain potential target size effects (or lack thereof). 

Although researchers have argued that motivation plays a mediating role in success-manipulation effects, it is unclear how motivation affects practice within a practice session, other than affecting participants’ effort in the task. Changes in effort alongside such success manipulations have not been examined. Moreover, in studies where success manipulations have resulted in consolidation benefits [12,13], somewhat artificial laboratory tasks have been used, which would force individuals to become reliant on augmented feedback to succeed and/or adopt certain control strategies. For example, in performing a coincident anticipation-timing task with stringent (versus lax) timing goal feedback, within milliseconds, performers would come to rely on the extrinsic feedback at the expense of their own intrinsic feedback about errors [12]. Therefore, there are issues with the generalisability of results from these tasks as a result of the asymmetries between strategies adopted in practice and tested in retention, which may be independent of success perceptions.

### 1.4. Aims of Present Study 

Based on the mixed evidence with regard to the efficacy of success manipulations on motor learning and issues concerning how it works, in the current study, we addressed some potential limitations with previous methods. Our aims were to reproduce the learning benefits that have been reported with success-enhancing manipulations (i.e., different target sizes) and study how such manipulations impact on processes related to learning by adopting various process measures. A dart-throwing task was chosen to address these aims, and we measured: (i) time between throws to index processing and planning activities, (ii) movement kinematics to alert us to changes in movement velocity and amplitude related to target size, and (iii) indices of effort. The effort-related indices included psychophysiological measures of arousal, as assessed through electrodermal activity (EDA), as well as physical effort, as measured through muscle co-contraction and EMG (electromyography). 

### 1.5. Hypotheses

#### 1.5.1. Behavioural and Psychological Measures

If enhanced success perceptions are beneficial to motor learning, we would expect a group practicing throwing to a large target (Large-target group) to be more accurate than a group practicing throwing to a small target (Small-target group) in retention. Differences only in retention (and not in practice) would lead to inferences that memory consolidation processes were involved. Rather than just measuring overall success, we studied outcome expectation, since reward prediction error-related and reinforcement learning-related dopaminergic signals in response to unexpected positive outcomes has been evidenced in neurophysiological research [23,24]. Although a small-target group should experience lower success than a large-target group, the small-target group should have more unexpected success (and hence reward), as noted on the occasional trials where expected performance is surpassed. This might be a reason for a lack of group differences in some research. Therefore, outcome expectation error was collected, along with measures of self-efficacy and perceived competency, as indicators of success perceptions. Target size was also expected to impact consistency in throwing, with more between-trial variability in the small-target group versus the large-target group during practice, as a result of the higher number of target misses that would require correction. 

#### 1.5.2. Transfer and Process Measures

Manipulations to target size might affect how the action is executed in practice, leading to a stiffer, less efficient pattern of activation in the muscles of the throwing arm for the small-target group rather than the large-target group [25]. Kinematic measures of shoulder and elbow angles, angular velocity, and temporal markers of preparation and end of throw were also assessed to alert to differences in control strategies. For a proxy measure of effort and arousal during practice, we collected psychophysiological measures of EDA [26,27]. If participants were feeling more motivated during practice as a result of the greater success, then the large-target group might show higher levels of arousal in general than the small-target group. 

In transfer tests at the end of retention, participants first performed a secondary tone-counting task whilst throwing. The added demands of a secondary cognitive task were expected to interfere more with performance for the small rather than the large-target group, if practice with a smaller target promoted more explicit learning (i.e., greater conscious control and cognitive processing, leading to a reduced capacity for additional cognitive tasks). The small-target group was also expected to report more explicit rules regarding how to throw than the large-target group, due to the greater propensity for the learners to engage in error-correction processes and test relevant hypotheses to improve their task performance [14]. 

In other transfer tests, participants predicted the landing zones of each throw (in the absence of vision) to assess whether either target practice had resulted in an improved awareness of outcome error (i.e., an index of intrinsic feedback processing). The small-target group was expected to be more accurate due to the need for more error processing in practice. Participants were also asked to regulate the pace of their throws to a slow or fast-paced metronome. In the fast-paced condition, the time between a set of throws was short, limiting feedback processing or movement planning. Practice throwing to a smaller target should lead to slower-paced throws, due to additional evaluation and planning-related demands. Hence, this fast-paced condition in transfer should lead to more errors for the small-target group, whereas the large-target group would show costs as a result of slowing down, if this is one of the reasons why learning differences between target sizes are seen.

## 2. Materials and Methods

### 2.1. Participants and Groups

Right-hand dominant women (*M* age: 21.4 year; 18–31 years) with normal or corrected vision and who were free from upper-limb injuries or neurological disorders were recruited via online advertisements. Participants had not played darts more than five times ever or more than once in the past 12 months, and they were not a competitive athlete in any throwing sport. We chose to restrict only to women participants, because in general, they have had less experience with darts than men, making it easier to recruit and reducing potential individual difference factors that can decrease power in such between participant designs. All the participants gave informed consent in accordance with the ethical procedures of the university, and then were randomly assigned to either a Large-target (*n* = 14) or Small-target group (*n* = 15). Remuneration of $11/h was paid to participants.

Three participants were initially excluded from analyses due to large errors on either the pretest or the first block of acquisition (≥2 *SD*s above group mean). Since these were identified early, participants were replaced until there were *n* = 15/group. Later analysis of individual acquisition and retention data showed that one participant neither improved in outcome accuracy during practice (comparing Block 1 to Block 10), nor exhibited learning as determined from a comparison of the initial block of acquisition to retention. Subsequently, this participant was excluded from analyses resulting in *n* = 14 in the Large-target group.

### 2.2. Task and Apparatus

We employed a dart-throwing task that was a simplified version of a task used in a previous study [21]. The throwing distance was decreased (200 cm from the dart board), and there were no horizontal accuracy requirements. Participants were only asked to hit a large (10-cm height) or small target band (2-cm height) that was displayed on a 56-cm wide posterboard overlaying a bristle dartboard. The task was simplified in an effort to reduce performance variability (through a reduction in force and the associated force variability on a shorter throwing distance [28]). By reducing variability, we hoped to increase the power to detect effects should they exist, and improve the likelihood that participants were practising at performance saturation (or asymptote), which has been shown to enhance memory consolidation in other cognitive and visual motor tasks [29]. Based on pilot testing, our target bands were chosen to better match the success rates experienced in previous research where learning-related group differences have been noted (i.e., ~50% for the easy group and less than a 10% success rate for the difficult group [12,13]). If the difficult target is only of moderate difficulty, success rates might not be sufficiently depressed. Conversely, if the easy target is too easy (closer to 100% success), it will lack challenge, and success would not be internalised. 

**Figure 1:**
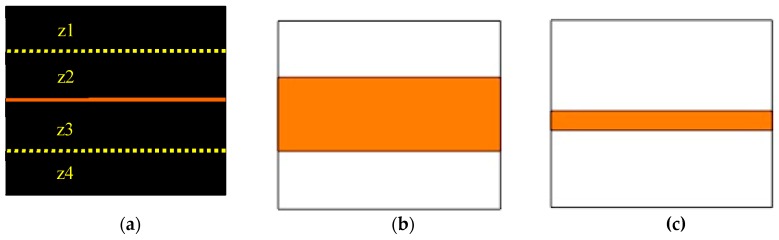
Schematic of the dartboard with target(s): (**a**) horizontal line target (solid orange) during pretest and Day 2’s tests of learning; zones z1–z4 are denoted in reference to the prediction tasks on Day 2, (**b**) large horizontal band target presented to the Large-target group, and (**c**) small horizontal band target presented to the Small-target group during acquisition.

In the pretest (Day 1), and retention or transfer testing (Day 2), participants aimed at a solid horizontal line that depicted the target (Figure 1a). During acquisition, participants aimed at either a large or small horizontal band. The targets were drawn on rectangular (56 × 71cm) posterboards that were affixed over a bristle dartboard that was surrounded by thick Styrofoam (see Figure 1b,c). Midlines of the horizontal target bands were parallel to the floor, at a height of 173 cm.

Three darts (26 g) were used to perform the throwing task. On some throws, participants wore a pair of liquid crystal, visual occlusion goggles (Translucent Technologies Inc., Toronto, Ontario, Canada) that were activated via a custom hand-held switch. A video camera was set up behind and above the participant so that dart landings were recorded and later analysed for positional, vertical error. A second video camera recorded the participants’ throwing motion from the right side (sagittal plane) at 30 frames/s. 

To obtain a measure of arousal, two disposable pre-gelled Ag/AgCl electrodes (0.5% chloride salt; Biopac systems Inc.) were affixed to the thenar and hypothenar prominence on the participants’ non-dominant palm for the recording of EDA. Exosomatic EDA was measured with a constant current of 0.5 V. Two EMG electrodes were attached to each belly of the tricep and bicep muscles of the throwing arm, and a grounding electrode on the acromion process was used to measure muscular activity. Both EMG and EDA signals were wirelessly amplified and transmitted to a data acquisition module unit (BioNomadix MP150, Biopac systems Inc., Montreal, Quebec, Canada) and recorded at a sampling rate of 1 KHz for post-experiment analyses (AcqKnowledge 4.2; Biopac systems Inc., Montreal, Quebec, Canada).

Participants completed the perceived competency and task interest/enjoyment subscales of the Intrinsic Motivation Inventory (IMI; [30]), the intrinsic motivation and amotivation subscales of the Situational Motivation Scale (SMS; [31]), and three customised items concerning whether they: (1) looked forward to practising a similar throwing/aiming activity in the future, (2) were motivated to do well during practice, and (3) felt successful during practice. All the questionnaires were scored on a seven-point Likert scale (1 = “strongly disagree”, 7 = “strongly agree”). During practice, participants reported self-efficacy in making at least one “hit” out of the next three throws. This was assessed using a self-efficacy scale that ranged from 0% to 100% in increments of 10, with descriptors, “0” = “not at all sure”, “10” = “not sure”, “40” = “somewhat sure”, “70” = “pretty sure”, and “100” = “very sure” [32].

### 2.3. Procedure

The study was conducted over two sessions separated by ~24 h (see Table 1). On Day 1, there was a pretest, followed by an acquisition (or practice) phase. Before the pretest, participants completed only the perceived competency subscale of the IMI and were fitted with EDA and EMG electrodes and transmitters. Reflective stickers were attached to the acromion process of the scapula (shoulder), the lateral epicondyle of the humerus (elbow), the styloid process of the ulna (wrist), and the first knuckle of the index finger of the throwing arm for post-experiment video analysis of throwing kinematics. Then, participants stood still for one minute while EDA and EMG baselines were recorded. Instructions were also given on the task, including a demonstration on how their throwing motion was to be maintained in the sagittal plane and where the dart should be gripped. 

#### 2.3.1. Pre-Test

Participants performed a warm-up throw to familiarise them with the weight of the dart and test out the general instructions (outcome was not recorded). Then, there followed six no-vision pretest trials. When participants pulled back their wrist (elbow in maximum flexion) and were about to throw, the goggles were manually activated to turn opaque, preventing vision of the throw’s outcome and hence error feedback. Vision was restored between throws once the outcome of the dart’s landing position was recorded by the experimenter and the dart was removed. Inter-trial intervals for no vision trials were not recorded. 

#### 2.3.2. Acquisition

Participants were instructed to make as many “hits” as they could. A “hit” was recorded when a dart landed within the horizontal band (regardless of its position on the *x*-axis). Three darts were placed on a stool to the right and slightly behind the participant and they were asked to retrieve and throw each dart one at a time. After each throw, participants were asked to turn and reach for a dart before looking back at the target again to make the next throw. This was a necessary step in measuring pre-throw duration. Pre-throw duration was the first moment when participants fixated on the target before they began a throw, to the moment when the dart was released. In between sets of three trials, participants tallied their hits and misses on a score sheet to help make successes (or failures) salient, and the experimenter returned the darts. 

Ninety acquisition trials were performed. Before trials 4, 31, and 61 (trial 4 was chosen as participants were less likely to be overwhelmed by initial instructions), participants’ self-efficacy and outcome expectations were probed. The self-efficacy rating scale was posted to the right of the dartboard, and participants were prompted for their self-efficacy on making at least one hit out of the next three throws (0% to 100%), followed by their expected outcome or number of hits (zero to three). At the end of acquisition, participants filled out the IMI and SMS subscales and responded to the three customised questions probing motivation and success. Then, they were reminded to get sufficient rest and avoid playing darts before the next session.

#### 2.3.3. Delayed Tests of Learning

On Day 2, participants completed only the perceived competency subscale of the IMI before retention and transfer. One warm-up throw was performed before six no-vision retention trials. Then, other tests were conducted consisting of nine trials per condition. First, a full-vision retention test was conducted, followed by an outcome prediction test (no-vision). In this latter condition, participants wore occlusion goggles, and after each trial, predicted the landing zone of the dart (no feedback was given; see Figure 1a). Then, a dual-task, tone-counting condition was performed. Participants completed three sets of three throws while mentally counting the number of high-pitch tones that were played in a sequence of high and low tones (300 ms/tone), interspersed by intervals of 500 to 1000 ms. Three unique random sequences were generated for each set. Participants did not begin throwing until after the first tone of the sequence was played. Subsequent darts for the set were handed to participants two to three seconds after they had thrown the previous dart, so as to regulate throw pace, ensuring that the number of tones were approximately matched across participants. In the final tests, participants threw darts at both a fast and slow pace, which were counterbalanced for order. Pacing was achieved through auditory tones, which dictated when the next dart should be thrown. Pilot work showed that the fast pace was the minimum amount of time needed to grip and throw the dart with some accuracy (one tone/s), whereas the slow pace was long enough so that participants could evaluate the outcome and make any new plans or adjustments between throws (one tone/five seconds). 

At the end of the study, participants were asked to report any task-relevant rules, techniques, or method that they had generated or become aware of during acquisition on Day 1. Participants were paid (~$20 Cdn; they were paid an hourly rate and testing took between 1.5–2 h) and debriefed.

### 2.4. Data Collection and Analysis

#### 2.4.1. Outcome Variables

Accuracy was derived post-test from videos recorded of the dartboard and completed with Dartfish © Prosuite video analysis software (Dartfish, Alpharetta, GA, USA). The positional error of the dart landings, as measured in the vertical, *y*-dimension from the target line (used in the pretests, retention tests, and transfer tests) or from the midline of the horizontal target bands (acquisition) was determined. For acquisition, the positional error was determined from the first and last nine trials of practice (even though measures of successful target hits were noted for all trials). From these positional errors, constant error (CE), absolute error (AE), and variable error (VE) were derived for each block of nine trials. The mean CE, AE, and VE across blocks or time were compared between groups in Group (Large-target versus Small-target) × Time, repeated measures (RM) ANOVAs. Separate analyses were conducted on pretests, acquisition, and retention, although we did compare the no-vision pretest to the no-vision retention test in a 2 Group × 2 Time RM ANOVA. For retention, accuracy on both vision and no-vision retention tests were compared in a 2 Group × 2 Vision RM ANOVA. Independent t-tests were used to compare across groups in the pretest and other transfer tests on Day 2. 

Target hits during acquisition were analysed in blocks of 30 trials (i.e., mega-block) and analysed in a 2 Group × 3 Mega-block RM ANOVA. For non-parametric data, such as the accuracy of tone-counting responses in the secondary and outcome-prediction tasks, group differences were analysed with the Mann–Whitney U-test.

#### 2.4.2. Measures of Success, Competence, and Motivation

Self-efficacy ratings were analysed in a 2 Group × 3 Mega-block (self-efficacy obtained at the beginning of each 30-trial Mega-block) RM ANOVA (one participant in the Large-target group did not provide self-efficacy ratings for Block 1 and was excluded from RM analyses). Outcome expectation was compared with actual performance on each of three probes. For each participant, they either performed below, at, or above expected levels. These frequencies, for each Mega-block, were compared across groups using Fisher’s exact test. Ratings of perceived competency, interest/enjoyment, motivation, and success were analysed with the Mann–Whitney U test.

#### 2.4.3. Process and Motor-Control Measures

EDA, EMG, kinematic, and trial movement duration data were analysed for all participants for blocks 2 (t10–18), 5 (t37–45), and 8 (t64–67) in practice. Three participants were excluded from analyses due to data collection errors (*n* = 2 Large-target, *n* = 1 Small-target). The EDA (in μS) waveform was re-sampled at 62.5 Hz, median-smoothed on 62 samples, and low-pass filtered at 1 Hz to provide the skin conductance level (SCL) for each trial. Pre-throw EDA was collected in the duration between target fixation and dart release. Post-throw EDA was from dart release to the next trial target fixation. For the last trial of each set of three throws, EDA duration was estimated using the post-throw duration of the previous trial. Each pre-throw and post-throw was subtracted from the baseline (quiet standing) mean EDA for each participant and converted to *z* scores. The *z*-transformed EDA data (in *SD* units) were analysed in a 2 Group × 3 Block (2, 5, and 8) × 2 Throw Period (pre and post) RM ANOVA.

For EMG, we took a measure of the co-contraction of the major upper arm muscles to provide a proxy of physical effort when throwing. The waveform was first band-pass filtered (5 and 500 Hz) and transformed to root mean square (RMS), using 10-ms windows [33]. The mean and max RMS EMG were derived for the tricep and bicep muscles for all trials in acquisition blocks 2, 5, and 8. Muscular activity (mV) was analysed between the onset of the muscle to the moment of dart release. Onset was defined as the point of earliest continuously rising RMS EMG deviating above baseline. Normalised mean RMS EMG (Norm–Mean) percentages were obtained by dividing the mean RMS EMG by the maximum tricep’s RMS EMG for each trial. The ratio of Norm–Mean triceps to biceps activity (i.e., co-contraction) for a trial was calculated and analysed in a 2 Group × 3 Block RM ANOVAs on the same three blocks of acquisition trials as the EDA data.

Shoulder and elbow joint angles were determined from video using Dartfish © Prosuite (Dartfish, Alpharetta, GA, USA), based on methods described in [25]. Each of these angles was tabulated at two moments for each trial, first at the moment of maximum retraction before dart release and then at the moment of dart release. The amplitude of joint movement (degrees), movement time (*ms*), and angular velocity were calculated based on these two points. Standard deviations of joint amplitude, movement time, and angular velocity were also calculated to provide a measure of movement variability for each block. Block means and *SD*s were independently analysed in 2 Group × 3 Block (2, 5 and 8) RM ANOVAs.

Pre-throw duration was the time between target fixation and dart release. Post-throw duration was the time between dart release and target fixation for the next throw. When participants did not take their eyes off the target or were still moving towards the throw line while their eyes were on the target, the target fixation time was marked as the first frame where participants stopped moving. For the last trial of each set, post-throw duration was not analysed. These duration measures were analysed in a 2 Group × 3 Block × 2 Time Period (pre-throw and post-throw) RM ANOVA on the same three blocks of acquisition as EDA and EMG.

Finally, the number of rules or strategies generated was analysed as a function of group, using the non-parametric Mann–Whitney *U*-test. The correlation coefficient, *r*, was reported as the effect size for Mann–Whitney *U*-tests. Cramer’s Phi (*φ*) was reported as effect size for Fisher’s exact test. For parametric data, partial eta squared (*η^2^_p_*) values are reported as measures of effect size, and post-hoc analyses were conducted using Tukey’s honestly significant difference test (*p* < 0.05) for all significant effects. Greenhouse–Geisser corrections were applied for violations to sphericity. Where t-tests were performed, Cohen’s *d* is reported.

## 3. Results

Descriptive statistics for each measure and phase for all the main effects (and significant group-related interaction effects) are detailed in Table 2. All the test statistics for significant effects, or those where *F* >1.5 or *t* >1, are given in Table 3.

### 3.1. Success Rates and Success Perceptions Were Affected by Target Size Manipulations

**Figure 2:**
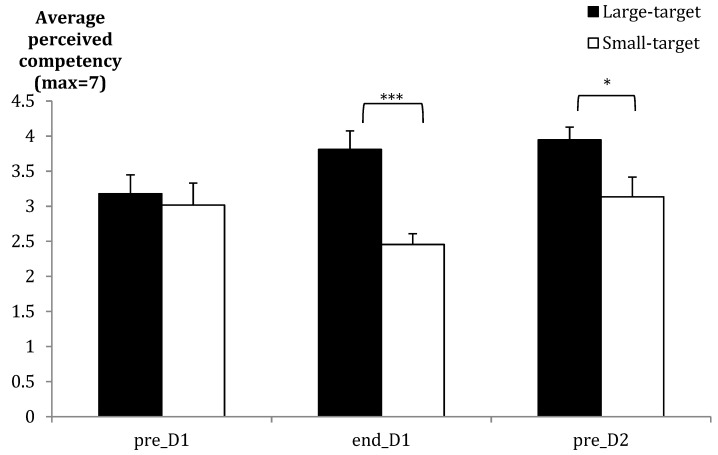
Mean group ratings (SE bars) for perceived competency before Acquisition Day 1 (pre_D1), after Acquisition Day 1 (end_D1), and before retention tests on Day 2 (pre_D2). * *p* < 0.05; *** *p* < 0.001.

**Figure 3:**
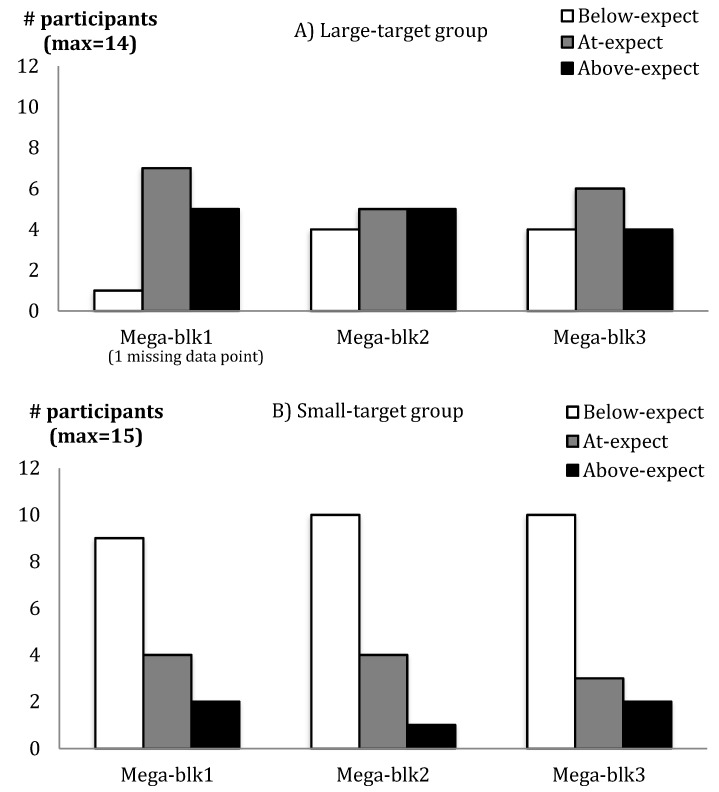
Number of participants performing below expectations (Below-expect), at expectations (At-expect), and above expectations (Above-expect) in the (**A**) Large-target and (**B**) Small-target groups as a function of Mega-blocks (Mega-blk) of 30 trials across practice.

The Large-target group had more hits in practice, and reported higher self-efficacy, success, and competency. The competency data are illustrated in Figure 2 where group differences are seen at the end of practice and before retention. A practice block interaction for self-efficacy probes was due primarily to a larger change between the first and second self-efficacy probes for the Large-target group (*M*_change_ = 25) compared to the Small-target group (*M*_change_ = 10). Both groups were significantly more confident on the final self-efficacy probe compared to the first (see Table 2). The Large-target group also showed a mix of expectations regarding their expected success compared to the Small-target group, which mostly performed worse than expected (see Figure 3A,B). Based on Fisher’s exact test on each Mega-block, significant group differences were only seen for Block 1.

### 3.2. Success Rates and Perceptions Did not Impact Outcome Measures

#### 3.2.1. No Vision Pre-Test

**Figure 4:**
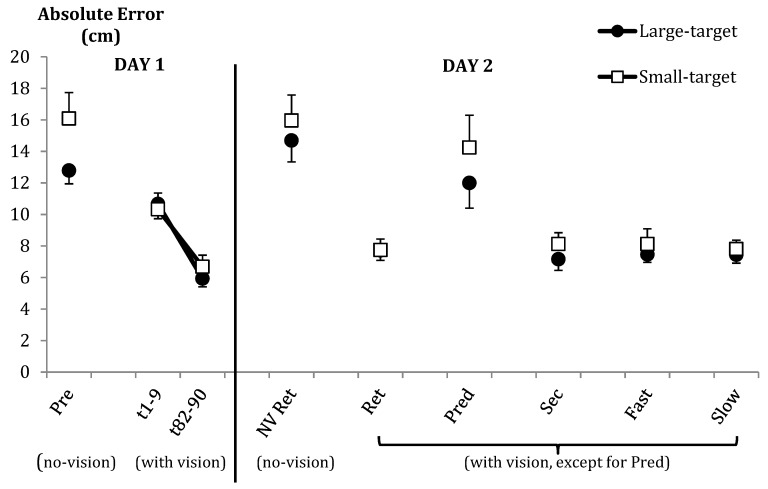
Mean absolute error (and SE bars) as a function of testing phase and condition. Pre = No-vision pretest; t1–9 = First nine acquisition trials; t82–90 = Final nine acquisition trials; NV Ret = No-vision retention; Ret = Retention; Pred = Outcome prediction test (no vision), Sec = Secondary task test; Fast = Fast-paced test, and Slow = Slow-paced test.

Absolute error (AE) data are illustrated for all phases of the experiment in Figure 4. Unless otherwise indicated, constant error (CE) displayed the same trends as AE throughout the study. There were no group-related differences in either AE or variable error (VE).

#### 3.2.2. Acquisition

Performance improved from early (Block 1) to late acquisition (Block 10) for all measures of outcome accuracy and consistency, but there were no group-related effects (for all measures, Fs <1 for all group-related effects). For illustration of changes in error across time, see Figure 4. 

#### 3.2.3. Retention Tests

The groups did not differ in any measure of accuracy in retention when comparing them across the no-vision and full-vision retention tests (all Fs <1; see Figure 4). Not surprisingly, there was an effect of vision, with lower AE when throwing with vision than without (similar vision effects were also seen in CE and VE; see Table 3). Comparisons of the no-vision pretest with the no vision retention test also did not result in any group-related effects.

#### 3.2.4. Other Delayed Tests of Learning

In other tests of learning, there were generally no group differences in either accuracy or consistency (see Figure 4). In addition to a general lack of group differences in outcomes during the prediction test, there was no significant group difference in prediction response accuracy. On average, the groups made approximately 5.7 errors (max = 9), when predicting landing zones, indicating an accuracy of ~37% (chance = 25%). No differences were noted in outcome error under secondary task conditions, nor were there differences in the accuracy of responses in this condition. Changes to the pacing of throws did not impact accuracy or result in predicted group differences in retention.

### 3.3. Success Did Not Impact Motivation

The groups were not significantly different regarding measures of intrinsic motivation, amotivation, other customised motivational items, and interest/task enjoyment subscales.

### 3.4. Processing Durations and Movement Kinematics Were Affected by Target Size Manipulation

#### 3.4.1. Pre-Throw and Post-Throw Duration

**Figure 5:**
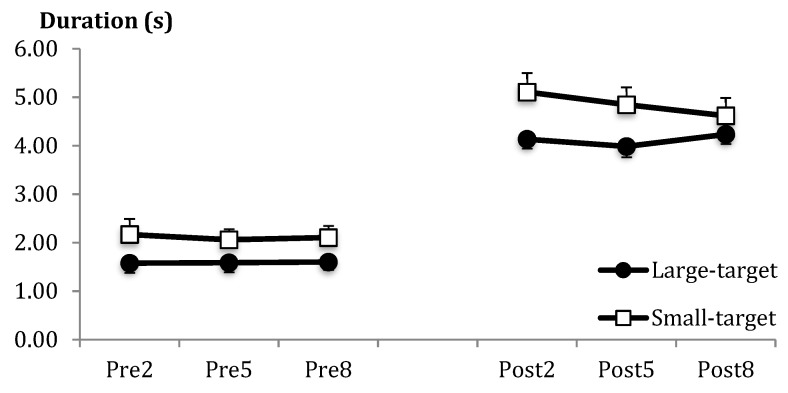
Mean duration (and SE bars) as a function of pre-throw (Pre) and post-throw (Post) periods for blocks 2, 5, and 8.

The Small-target group was significantly slower overall than the Large-target group (see Figure 5). Although post-throw duration was longer than pre-throw duration, there were no other effects involving group.

#### 3.4.2. Explicit Knowledge and Strategies

There were no group differences.

#### 3.4.3. EDA (Electrodermal), EMG (Electromyography), and Joint Kinematics

Analysis of the *z*-transformed means for EDA difference scores in relation to baseline showed only a significant effect of throw period. The post-throw period had larger EDA difference score values than the pre-throw period. No other EDA effects were significant, nor was there any indication that there was more co-contraction in the EMG activity for the Small-target group versus the Large-target group during practice.

**Figure 6:**
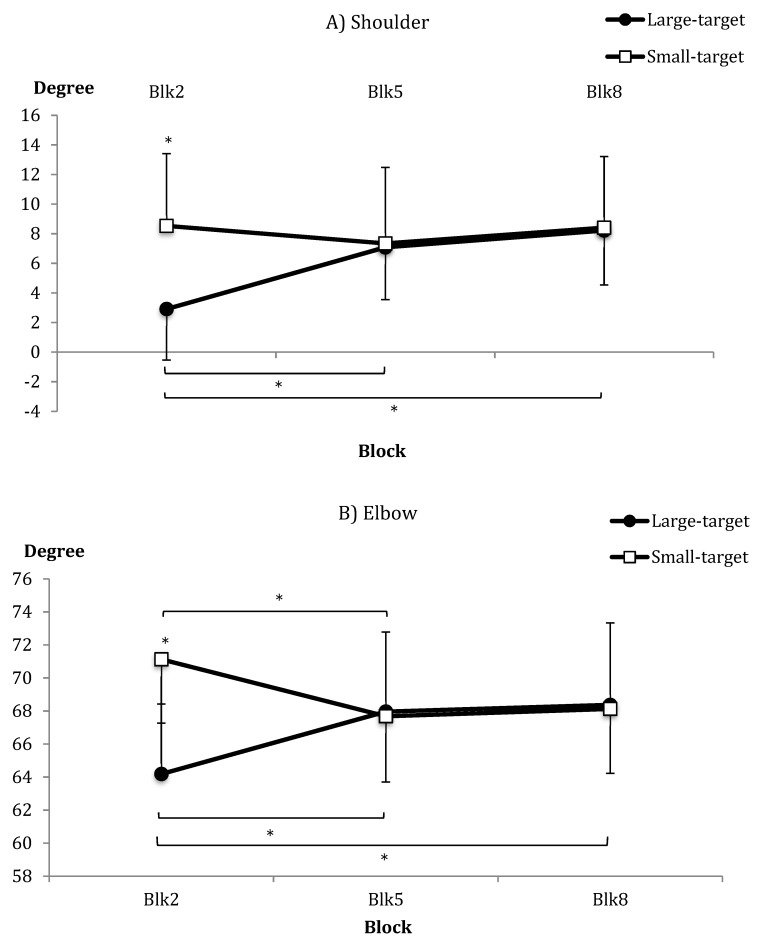
Mean (**A**) shoulder and (**B**) elbow joint amplitudes (and SE bars) as a function of group and block (blocks 2, 5, and 8). * *p* < 0.05.

With respect to kinematics, no significant group differences were shown in mean angular velocity and movement time and their *SD*s. For joint amplitude, the groups were not different overall for either shoulder amplitude or elbow amplitude, and only the shoulder amplitude differed across blocks, indicating an increase in amplitude over practice. There were significant group × block interactions for both shoulder and elbow amplitude (see Figure 6A,B, respectively). Post-hoc analyses indicated group differences only in the early practice block (Block 2), where amplitudes were larger for the Small-target group compared to the Large-target group for shoulder and elbow. The Large-target group showed an increase in shoulder and elbow amplitude from early (Block 2) to end of practice (Block 8; mean change, shoulder = 5.3°; elbow = 4.19°). The Small-target group either showed no change or a significant decrease in elbow amplitude from blocks 2 to 5; see Figure 6B).

Analyses of the between-trial *SD* of amplitudes revealed a significant group × block effect for shoulder amplitude only. Post-hoc tests showed smaller *SD*s for the Large-target group versus the Small-target group only in Block 2. Only the Small-target group decreased variability across practice.

## 4. Discussion

Our aim was to test for success-related influences on motor learning through manipulations to target size. We controlled for factors that may have accounted for differences in results across studies (related to target difficulty and rates of success) and included process measures to help determine how target size manipulations and success-related variables potentially impact motor learning and might explain any subsequent group differences (or lack of difference). 

As a result of the manipulation, the groups were significantly different in terms of success rates, self-efficacy, outcome expectation error, and perceived competency (both post-acquisition and before retention). The Large-target group was overall more successful and rated themselves as more successful than the Small-target group. Although there were improvements across trials and into retention for both groups (i.e., in all error measures), there were no group-related differences. These findings cast doubts on the efficacy of success manipulations to impact motor learning. Instead, these data are in keeping with a growing body of published work failing to show success-related differences in motor learning outcomes [19,20,21,34]. Being and/or perceiving oneself to be more successful in practice than another group does not seem to be a sufficient factor to bring about learning-related outcome differences.

Motor learning benefits associated with enhanced perceptions of success or expectancies, such as self-efficacy, have been reported elsewhere [9,12]. This has resulted in the proposal that there is a motivational basis for these success-related effects, in accordance with predictions of social cognitive theory [35]. It is thought that when individuals are highly self-efficacious, they tend to become more motivated and hence exert more time and effort towards performance [9,11,36]. This would be evidenced in enhanced performance during practice. However, our data do not show support for this process or the resulting effects. The more efficacious participants in the Large-target group did not show improved accuracy in practice compared to the less efficacious, Small-target group. Although an increase in success perceptions is typically associated in a causative manner with improved motor learning in the literature, there is reason to question this relationship. Pre-existing group differences in task ability might be one explanation for the early performance differences between groups, especially as there has been evidence of differences as early as the first practice block [9,10,11]. Although it is unlikely that individual differences in random allocation explain all the group differences seen in the literature, it is possible that they might explain some of the positive results. In at least two other motor learning studies, measures of motivation (which is expected to mediate success perception effects) were shown to be unrelated to retention outcomes [37,38].

Even when success manipulations impact self-efficacy and related measures of competency, efficacy perceptions may not influence motivation in a consistent way. One reason why this relationship between efficacy and motivation might be different to that proposed by social cognitive theory [35] is that here (and in other motor learning studies), we are dealing with short-term effects on relatively simple, laboratory-based tasks. We are not tapping into whether individuals are prepared to engage in more practice to improve, or engage in more quality practice, as opportunities are not provided to do this within the confines of the study. In the present study, participants in the Small-target group reported lower success perceptions than the Large-target group, but this did not extend to differences in motivation ratings (both groups scored high on all measures of motivation). Even if the groups had been different on measures of motivation, it is not clear how motivation, beyond exertion of greater effort during practice, would translate into enhanced learning without the option for more motivated participants to practice more. Being more motivated to be accurate at dart-throwing might make one more variable in movement kinematics, or result in increased time to plan movements, which has been taken as a proxy of effort in other studies [39,40]. However, none of these measures defined the Large-target group. Rather, the Small-target group made larger, more variable joint movements, and took longer to plan and process their throws. In summary, not only did we fail to show a relationship between self-efficacy and motivation, if pre-throw duration is interpreted as a marker of cognitive effort and planning, these results are the opposite to what would be predicted based on an effort-related motivation explanation (cf. [1]).

With respect to movement kinematics, larger shoulder and elbow amplitudes and more variable shoulder movements were made by the Small-target group compared to the Large-target group early in acquisition. This is indicative of differences in motor control strategies as a function of the task demands. Aiming at more difficult targets requires greater precision, effort, and error correction, which might result in increased between-trial movement variability to achieve success early in practice. In the absence of group-related differences in other kinematic measures of movement time and angular velocity, we would have expected greater movement amplitude and variability to result in more variable outcomes [22]. However, there was no evidence of group differences in measures of outcome accuracy. In short, although there were some effects of target size on measures of motor control, these were small and isolated to joint amplitudes (not movement time and velocity, as we might have expected for movements to different target sizes [41]). Moreover, differences in kinematics were not related to overall outcomes.

Taking into consideration other applied psychological perspectives, a non-positive relationship between success perceptions and motivation might appear less surprising. In control theories of self-regulation, a negative relationship is thought to exist between self-efficacy and performance [42]. When standards of performance are not met, individuals are expected to be motivated to reduce the discrepancy between performance outcome and criterion standards. As such, increased motivation would be expected in the smaller target or less successful group. Other situational and individual factors could also influence the impact of low success (high error) on effort and performance [43]. Individual personality traits such as high task or mastery orientation, or conscientiousness [44], could lead to maintained or greater effort despite a lack of success or positive feedback, especially when individuals perceive a task to be of high difficulty. Although we did not collect data on participants’ initial state of motivation (only post-practice motivation), it is probable that participants in our study were generally highly motivated individuals. All had been paid to participate and had taken the time to respond to adverts and come to the laboratory for the study. 

The assertion that motivation and effort underlie phenomena observed with success-related manipulations was also assessed through psychophysiological means. Indicators of arousal, such as heart rate and EDA (electrodermal activity), are used as indirect measures of the attentional or mental effort expended by individuals [27]. However, we did not find group-related EDA distinctions as a result of the differential success experienced by participants. The only significant effect involved the throw period, indicating greater EDA in the period after the dart was thrown rather than before. This may indicate greater effort in post-throw feedback processing. There were also no group differences in EMG measures related to muscle co-contraction, which was thought to reflect effort. In summary, although there was some indication of increased post-throw feedback processing for the Small-target group (as evidence by longer processing durations), there was no evidence that this disadvantaged learning as purported by implicit learning advocates [18]. Even when individuals were tested under secondary task conditions, any effects associated with the longer processing time failed to result in poorer performance for the Small-target group. 

We also measured success expectations, anticipating that perhaps the Small-target group may have experienced more unexpected reward when targets were hit than the Large-target group (which might explain any lack of group differences). However, more participants in the Large-target group performed above expectations in the first practice block, hence experiencing more unexpected success or reward compared to the Small-target group, who tended to perform below expectations. According to neurophysiological research related to reward prediction error and reinforcement learning, a stronger dopaminergic or reward signal should be associated with practice on the large target in this study [23,24]. Despite this, we still did not find a learning benefit to practice on the large compared to the small target.

Through two independent studies involving manipulations to target size in a discrete, dart-throwing task, we have failed to show supportive evidence that enhanced success perceptions or expectancies benefit motor learning (see also [21]). Although target size manipulation had shown the predicted impact on success perceptions in both studies, with the larger target group experiencing heightened self-efficacy, perceived competency, and success, indices associated with learning were not sensitive to success. In the current study, the target size impacted measures of movement planning and feedback processing (i.e., pre-throw and post-throw durations) and motor control measures associated with movement amplitude and variability, but as before, these factors did not translate to differences in behavioural outcomes.

In considering other reasons for the discrepancies between studies, it is important to acknowledge that our participants were privy to the actual dart landing position relative to the target centre (and not just target hits or misses). It is possible that this information was used to make strategic modifications to the next trial, regardless of target size. Indeed, the Large-target group was just as variable in their landing outcomes as the Small-target group. Contrary to predictions that enhanced success would produce heightened motivation, and vice versa, the Large-target group did not report higher motivation scores. Although this lack of difference in motivation between groups may be moderated by the saliency of the exact outcome error or potential ceilings in motivation due to participant payment or mastery orientation in learning goals, the absence of motivation-related differences might negate any potential group differences in learning. Finally, it may be that these success-related effects are small, and that to reliably detect such differences, well-powered studies are needed. This may require a team effort across multiple labs.

With a sizable number of studies in support of using success-related practice interventions in motor learning, practitioners may be tempted to take manipulations of success and positive feedback to the extreme. Doing so could lead to undesired consequences. We still do not know how motivation is influenced by changes to self-efficacy or expectancies. Other empirical evidence suggests that a lack of difficulty or challenge during practice and unwarranted praise or peer comparisons can promote complacency, disinterest, and an extinction of effort in learners. There is a strong need for researchers who have been studying the role of social–cognitive and affective variables on motor learning to accompany this research with theory-driven process measures to better isolate under what conditions feelings of success or heightened expectancies help promote motor learning.

## 5. Conclusions

Contrary to recent evidence in support of the use of success-related practice manipulations to enhance motor learning and performance, our results indicate that manipulations to success perceptions are not necessarily associated with changes in learners’ motivation or behavioural indices of learning and performance. While practice target size influenced success perceptions, movement amplitude (in early practice) and error processing duration, it did not differentiate motivation, throwing accuracy and consistency. We conclude that manipulations to success perceptions alone are insufficient to benefit motor learning.

## Figures and Tables

**Table 1:** Summary of procedures and dependent measures. IMI: Intrinsic Motivation Inventory, SMS: Situational Motivation Scale.

Task and Procedures (# Trials) Day 1 (# Trials)	Day 2 (# Trials)
1. Familiarisation (1)	1. Familiarisation (1)
2. No vision pre-test (6)	2. No vision retention (6)
3. Acquisition (90)	3. Vision retention (9)
	4. No vision plus 5. Prediction (9)
	6. Secondary task (9)
	7. Slowed + speeded (9 + 9)
**Dependent Measures**	
Behavioural:	
1. # Target hits and outcome error (absolute error, constant error, variable error)	
2. Secondary tone-counting response	
3. Outcome-prediction response	
Questionnaire:	
1. Self-efficacy rating: 3 × acquisition	
2. Outcome expectation: 3 × acquisition	
3. IMI (competency): Pre and post Day 1, Pre Day 2	
4. IMI (interest/enjoyment): Post-Day 1	
5. SMS: Post Day 1	
6. Success perception check: Post Day 1	
7. Custom motivation items: Post Day 1	
8. Explicit knowledge: Post Day 2	
Process:	
1. EMG (electromyography), EDA (electrodermal), kinematics	
2. Trial or movement durations	

**Table 2:** Means and standard deviations (SDs) for all measures as well as medians (mdns) for non-parametric data associated with all main-effect comparisons and statistically significant group-related post-hoc differences.

Measure	Phase/Condition	Mean (SD)	Mean (SD)	Mean (SD)
Target hits (%)	Acq	Large = 44.1 (7.8)	Small = 11.3 (3.6)	
		B1 = 25.5 (16.9)	B2 = 27.0 (17.7)	B3 = 28.7 (21.1)
Self-efficacy probe (%)	Acq	Large = 63.33 (13.22)	Small = 29.78 (19.59)	
		B1 = 32.32 (22.09)	B2 = 49.29 (26.76)	B3 = 55.36 (28.89)
	Acq (Large)	B1 = 45.8 (19.8)	B2 = 70.8 (13.8)	B3 = 75.4 (16.6)
	Acq (Small)	B1 = 20.7 (17.1)	B2 = 30.7 (20.4)	B3 = 38.0 (26.0)
Perceived competency (1–7 scale)	Start Acq	Large = 3.2 (1.0) (mdn = 3.1)	Small = 3.0 (1.2) (mdn = 3.3)	
	End Acq	Large = 3.8 (1.0) (mdn = 3.8)	Small = 2.5 (0.6) (mdn = 2.2)	
	Ret	Large = 3.9 (0.7) (mdn = 4.0)	Small = 3.1 (1.1) (mdn = 3.0)	
Perceived success (1–7 scale)	End Acq	Large = 4.6 (0.8) (mdn = 5)	Small = 3.5 (1.1) (mdn = 3)	
Interest/enjoyment (1–7 scale)	End Acq	Large = 5.6 (0.6) (mdn = 5.7)	Small = 5.7 (0.6) (mdn = 5.9)	
Intrinsic motivation (1–7 scale)	End Acq	Large = 5.6 (0.6) (mdn = 5.5)	Small = 5.5 (0.7) (mdn = 5.8)	
Customised motivation (further practice), 1–7 scale	End Acq	Large = 5.5 (1.3) (mdn = 6.0)	Small = 5.5 (1.0) (mdn = 5.0)	
Customised motivation (do well in practice), 1–7 scale	End Acq	Large = 5.6 (1.1) (mdn = 6.0)	Small = 6.3 (0.8) (mdn = 6.0)	
Amotivation (1–7 scale)	End Acq	Large = 2.6 (0.6) (mdn = 2.5)	Small = 2.4 (0.9) (mdn = 2.3)	
#Rules or strategies accumulated	End Ret	Large = 1.9 (1.1) (mdn = 2.0)	Small = 2.3 (1.1) (mdn = 2.0)	
Outcome error: Absolute error (cm)	Pre	Large = 12.79 (3.16)	Small = 16.08 (6.39)	
	Acq	Large = 8.30 (3.68)	Small = 8.51 (3.88)	
		B1 = 10.49 (3.71)	B10 = 6.33 (2.44)	
	Pre vs. Ret	Large = 13.74 (4.26)	Small = 16.02 (6.22)	
		Pre = 14.49 (5.28)	Ret = 15.35 (5.66)	
	Ret V vs. NV	Large = 11.20 (5.27)	Small = 11.85 (6.31)	
		Vis = 7.73 (2.49)	NV = 15.35 (5.66)	
	Ret (V)	Large = 7.72 (2.37)	Small = 7.74 (2.68)	
	Prediction	Large = 11.99 (5.98)	Small = 14.25 (7.94)	
	Secondary	Large = 7.16 (2.67)	Small = 8.12 (2.77)	
	Fast	Large = 7.46 (1.89)	Small = 8.13 (3.72)	
	Slow	Large = 7.44 (1.98)	Small = 7.81 (2.15)	
Constant error (cm)	Pre	Large = –10.08 (6.55)	Small = –15.04 (7.41)	
	Acq	Large = –4.64 (4.29)	Small = –5.38 (5.13)	
		B1 = –7.63 (4.84)	B10 = –2.41 (2.77)	
	Pre vs. Ret	Large = –11.48 (6.51) Pre = –12.64 (7.33)	Small = –14.64 (7.37) Ret = –13.59 (6.94)	
	Ret V vs. NV	Large = –8.34 (6.68) Vis = –4.68 (3.00)	Small = –9.87 (7.23) NV = –13.59 (6.94)	
	Ret (V)	Large = –3.81 (2.72)	Small = –5.49 (3.12)	
	Prediction	Large = –9.58 (8.52)	Small = –11.79 (10.45)	
	Secondary	Large = –2.56 (3.94)	Small = –4.91 (3.89)	
	Fast	Large = –1.02 (4.30)	Small = –3.80 (3.04)	
	Slow	Large = –4.37 (2.90)	Small = –3.86 (2.95)	
Variable error (cm)	Pre	Large = 9.05 (3.30)	Small = 9.30 (3.24)	
	Acq	Large = 8.61 (3.08)	Small = 8.38 (3.04)	
		B1 = 10.14 (2.34)	B10 = 6.84 (2.77)	
	Pre vs. Ret	Large = 9.34 (3.63)	Small = 10.03 (3.51)	
		Pre = 9.18 (3.21)	Ret = 10.21 (3.85)	
	Ret V vs. NV	Large = 9.01 (3.42)	Small = 9.41 (3.34)	
		Vis = 8.22 (2.45)	NV = 10.21 (3.85)	
	Ret (V)	Large = 8.39 (2.66)	Small = 8.07 (2.31)	
	Prediction	Large = 8.13 (3.39)	Small = 9.35 (4.06)	
	Secondary	Large = 8.07 (2.72)	Small = 7.78 (2.64)	
	Fast	Large = 7.92 (2.56)	Small = 8.40 (3.42)	
	Slow	Large = 7.58 (2.11)	Small = 8.00 (1.95)	
Processing duration (s)	Acq	Large = 2.85 (1.43)	Small = 3.48 (1.82)	
		B2 = 3.28 (1.79)	B5 = 3.14 (1.64)	B8 = 3.16 (1.61)
		PreT = 1.87 (0.86)	PostT = 4.51 (1.16)	
Joint amplitude	Acq	Large = 6.07 (12.19)	Small = 8.09 (18.04)	
(shoulder) (°)		B2 = 5.94 (15.63)	B5 = 7.22 (16.05)	B8 = 8.32 (15.48)
	Acq/B2	Large = 2.91 (11.94)	Small = 8.53 (18.26)	
Joint amplitude	Acq	Large = 66.84 (15.87)	Small = 68.98 (14.38)	
(elbow) (°)		B2 = 67.92 (14.70)	B5 = 67.81 (15.44)	B8 = 68.24 (15.52)
	Acq/B2	Large = 64.19 (14.69)	Small = 71.13 (14.46)	
SD joint amplitude	Acq	Large = 3.60 (1.93)	Small = 4.23 (2.43)	
(shoulder) (°)		B2 = 4.24 (2.53)	B5 = 3.76 (1.90)	B8 = 3.82 (2.25)
	Acq/B2	Large = 3.37 (1.91)	Small = 4.98 (2.81)	
	Acq/B8	Large = 4.12 (2.57)	Small = 3.56 (2.00)	
SD joint amplitude	Acq	Large = 7.80 (2.78)	Small = 7.16 (2.69)	
(elbow) (°)		B2 = 7.86 (2.67)	B5 = 7.65 (2.94)	B8 = 6.85 (2.58)
Movement time (ms)	Acq	Large = 199.1 (93.2)	Small = 186.5 (50.0)	
		B2 = 188.0 (64.6)	B5 = 192.5 (63.7)	B8 = 196.6 (90.1)
SD movement time	Acq	Large = 43.7 (72.8)	Small = 37.7 (33.2)	
(ms)		B2 = 37.7 (38.0)	B5 = 45.2 (71.8)	B8 = 38.5 (51.2)
Angular velocity (°/s)	Acq	Large = 366.8 (130.1)	Small = 371.4 (93.9)	
		B2 = 371.2 (112.5)	B5 = 365.0 (107.0)	B8 = 371.0 (118.6)
SD angular velocity	Acq	Large = 54.6 (25.9)	Small = 60.9 (28.0)	
(°/s)		B2 = 62.7 (25.7)	B5 = 56.0 (32.4)	B8 = 55.2 (22.5)
Electrodermal activity *	Acq	Large = 6.30 (1.93)	Small = 5.09 (2.10)	
(μS)		B2 = 5.63 (2.00)	B5 = 5.55 (1.96)	B8 = 5.75 (2.37)
		PreT = 5.54 (2.09)	PostT= 5.75 (2.13)	
Electromyography co-contraction (ratio)	Acq	Large = 1.62 (.88) B2 = 1.77 (.76)	Small = 1.92 (.72) B5 = 1.83 (.83)	B8 = 1.72 (.87)

Key: Pre = Pretest; Acq = Acquisition; Ret = Retention; B = Block; PreT = Pre-throw; PostT = Post-throw; NV = No vision; Vis = Vision; ^ negative values indicate dart landings below the midline; * = pre-transformed change from baseline values.

**Table 3:** Summary table of statistics (test statistic, *p*-value, and effect size, either partial-eta squared, Cramer’s *φ* or Cohen’s d) for all main effect and group-related interactions when Fs >1.5 or t >1.

Measures	Effects	Test Statistic	*p*-Value	Effect Size
**Target Hits:**				
Acq	Group	F(1,27) = 218.02	***	*η^2^_p_* = 0.89
	Mega-blk	F(2,54) = 1.61	0.21	*η^2^_p_* = 0.06
	Group × Mega-block	F(2,54) = 2.33	0.11	*η^2^_p_* = 0.08
**Self-Efficacy Probes:**				
Acq	Group	F(1,26) = 27.98	***	*η^2^_p_* = 0.52
	Mega-blk	F(2,52) = 30.90	***	*η^2^_p_* = 0.54
	Group × Mega-blk	F(2,52) = 3.32	*	*η^2^_p_* = 0.11
**Outcome Expectation Error:**				
Mega-Blk 1	Group	Fisher’s exact test	*	*φ* = 0.55
**Perceived Competency:**				
End Acq	Group	U = 22.50	***	*r* = 0.67
Ret	Group	U = 52.00	*	*r* = 0.43
**Perceived Success:**				
End Acq	Group	U = 43.50	**	*r* = 0.53
**Customised Motivation (Do Well in Practice):**				
End Acq	Group	U = 66.50	0.09	*r* = 0.33
**Absolute Error (AE):**				
Pre	Group	*t* (20.8) = 1.78	0.09	*d* = 0.65
Acq	Blk	F (1,27) = 47.26	***	*η^2^_p_* = 0.64
Ret	Vision	F (1,27) = 58.19	***	*η^2^_p_* = 0.68
Pre vs. NV Ret	Group	F (1,27) = 2.27	0.14	*η^2^_p_* = 0.08
**Constant Error (CE):**				
Pre	Group	*t* (27) = 1.90	0.07	*d* = 0.71
Pre vs. NV Ret	Group	F(1,27) = 2.66	0.12	*η^2^_p_* = 0.09
Acq Blk1	Group	*t* (27) = 1.11	0.28	*d* = 0.41
Acq	Blk	F(1,27) = 44.79	***	*η^2^_p_* = 0.62
	Group × Blk	F(1,27) = 2.60	0.12	*η^2^_p_* = 0.09
Ret	Vision	F (1,27) = 57.10	***	*η^2^_p_* = 0.68
Sec	Group	*t* (27) = 1.61	0.12	*d* = 0.60
Fast	Group	*t* (27) = 2.03	0.05	*d* = 0.75
**Variable Error (VE):**				
Acq Blk1	Group	*t* (27) = 1.53	0.14	*d* = 0.57
Acq	Blk	F (1,27) = 38.07	***	*η^2^_p_* = 0.59
	Group × Blk	F (1,27) = 3.96	0.06	*η^2^_p_* = 0.13
Ret	Vision	F (1,27) = 5.19	*	*η^2^_p_* = 0.16
**Processing Duration:**				
Acq	Group	F(1,24) = 4.83	*	*η^2^_p_* = 0.17
	Throw Period	F(1,24) = 126.09	***	*η^2^_p_* = 0.84
	Group × Blk	F(2,48) = 2.15	0.13	*η^2^_p_* = 0.08
	Group × Blk × Period	F(2,48) = 2.02	0.14	*η^2^_p_* = 0.08
**Joint Amplitudes:**				
Shoulder	Blk	F(2,48) = 4.29	*	*η^2^_p_* = 0.15
	Group × Blk	F(2,48) = 6.14	**	*η^2^_p_* = 0.20
Elbow	Group × Blk	F(2,48) = 6.94	**	*η^2^_p_* = 0.22
**Variability (*SD*) of Joint Amplitudes:**				
Shoulder	Group × Blk	F(2,48) = 4.43	*	*η^2^_p_* = 0.16
Elbow	Blk	F(2,48) = 1.54	0.23	*η^2^_p_* = 0.06
**Electrodermal Activity (EDA):**				
Acq	Period	F(1,24) = 15.24	**	*η^2^_p_* = 0.39
Acq	Group × Period	F(1,24) = 3.46	0.08	*η^2^_p_* = 0.13

Key: Pre = Pretest; Acq = Acquisition; Ret = Retention; Sec = Secondary task test; Blk = Block; Fast = Fast-paced test; NV = No vision; * *p* < 0.05; ** *p* < 0.01; *** *p* < 0.001.
